# Demonstration of Clinical Meaningfulness of the Integrated Alzheimer’s Disease Rating Scale (iADRS): Association Between Change in iADRS Scores and Patient and Caregiver Health Outcomes

**DOI:** 10.3233/JAD-220303

**Published:** 2022-07-19

**Authors:** Alette M. Wessels, Mark Belger, Joseph A. Johnston, Youying Yu, Dorene M. Rentz, Sherie A. Dowsett, Julie Chandler

**Affiliations:** a Eli Lilly and Company, Indianapolis, IN, USA; b Department of Neurology, Massachusetts General Hospital, Harvard Medical School, Boston, MA, USA; c Center for Alzheimer Research and Treatment, Department of Neurology, Brigham and Women’s Hospital, Harvard Medical School, Boston, MA, USA

**Keywords:** Alzheimer’s disease, care burden, global burden of disease, outcome assessment (health care), outcome measures, patient relevant outcome

## Abstract

**Background:**

The integrated Alzheimer’s Disease Rating Scale (iADRS) is a validated cognitive/functional composite that effectively captures cognitive and functional decline over a broad spectrum of disease. The clinical meaningfulness of change on iADRS can be supported by establishing an association with changes on important health outcome measures.

**Objective:**

To evaluate the relationship between change on the iADRS and changes in health outcomes in individuals with mild cognitive impairment (MCI) due to Alzheimer’s disease (AD), or mild or moderate AD dementia using placebo data from four AD clinical trials and data from one AD observational study.

**Methods:**

Analysis of covariate (ANCOVA) models were used to estimate the relationship between 18-month change on the iADRS and changes on health outcome measures (related to cost, quality of life, and caregiver burden). The regression coefficients for the iADRS were used to compute impact of natural disease progression and disease-modifying treatment on health outcomes. Additional ANCOVAs were conducted to understand whether cognition and/or function was the underlying explanation of any association between iADRS and health outcome change.

**Results:**

Across datasets and disease stages, a worsening on the iADRS was significantly associated with increased societal costs, caregiver burden (time and distress) and worsening in measures of patient quality of life.

**Conclusion:**

Decline on the iADRS was associated with worsening in health outcome measures. These findings suggest that the iADRS can be used in clinical trials as a proxy measure of clinically meaningful outcomes of AD progression.

## INTRODUCTION

In the study of Alzheimer’s disease (AD) in the clinical trial setting, clinical outcome assessments need to be appropriate for the population under study. To optimally study the efficacy of disease-modifying therapies, identifying a clinical assessment outcome that captures the core aspects of AD (i.e., decline in cognition and function) and is sensitive, responsive, and able to detect clinically meaningful changes across the disease continuum is important.

The integrated Alzheimer’s Disease Rating Scale (iADRS) is a cognitive/functional composite of two widely accepted measures, the Alzheimer’s Disease Assessment Scale - Cognitive Subscale 13-item version (ADAS-Cog_13_) and the Alzheimer’s Disease Cooperative Study - instrumental activities of daily living (ADCS-iADL) scale. It has been validated, with well-described statistical properties [1–3], and has been used, and is currently in use, as a clinical outcome measure in Phase II and III clinical trials in AD [4–11]. Data from the validation study [1], as well as clinical trials, demonstrated that the iADRS was effective in capturing clinical progression from mild cognitive impairment (MCI) due to AD through moderate AD dementia, as well as treatment effects across the early disease spectrum.

Establishing whether a change in an outcome is clinically meaningful can be accomplished using various methodologies and it is prudent to weigh the evidence from more than one approach. Cognitive decline and impairment in activities of daily living have considerable impact on the individual and their caregiver, as well as on health care and community care costs [12]; accordingly, patient quality of life, caregiver burden (including time commitment), and costs are important outcomes in the AD realm [13–15]. An association between changes on the iADRS and changes in health outcome measures would provide evidence for iADRS changes being considered as clinically meaningful.

In this paper, retrospective analyses were conducted to evaluate the relationship between change on the iADRS and changes in health outcome measures (patient quality of life, caregiver burden, and costs) in individuals with MCI due to AD, or mild or moderate AD dementia. Where associations between changes on the iADRS and health outcomes measures were evident, the potential impact of a disease-modifying treatment on these changes was evaluated.

## METHODS

### Databases used in analyses and their study designs

Datasets that included findings from the iADRS (or the components of iADRS) and that were accessible to the authors were included. Overall, data from five studies were included: one observational study (GERAS) and four randomized clinical trials (EXPEDITION-1, EXPEDITION-2, EXPEDI-TION-3, and AMARANTH). For the randomized clinical trials, only placebo data were included. As in prior publications [16], data from EXPEDITION-1 and -2 were pooled (EXP1+2) since the study designs were identical. The design of each study is briefly described in Table 1 [5, 6, 17–22].

**Table 1:** Studies included in the analyses [5, 6, 16–22]

	AMARANTH	EXPEDITION-1 and -2 (Pooled data)	EXPEDTION-3	GERAS
Study Design	Phase 2/3 lanabecestat treatment trial	Phase 3 solanezumab treatment trials	Phase 3 solanezumab treatment trial	Prospective, observational study
NCT number	NCT02245737	NCT00905372 and NCT00904683, respectively	NCT01900665	–
Aim	Primary outcome - change from baseline to Week 104 in ADAS-Cog_13_ score	Primary outcomes - change from baseline to Week 80 in ADAS-Cog_11_ and ADCS-ADL scores for EXPEDITION-1; ADAS-Cog_14_ for EXPEDITION-2	Primary outcome - change from baseline to Week 80 in ADAS-Cog_14_ score	Assess resource use and costs associated with AD for community-dwelling patients and caregivers
Enrollment countries	Australia, Belgium, Canada, France, Germany, Hungary, Italy, Japan, Korea, Poland, Puerto Rico, Romania, Spain, UK, USA	Argentina, Australia, Brazil, Canada, France, Germany, Italy, Korea, Japan, Poland, Russian Federation, Spain, Sweden, Taiwan, UK, USA	Australia, Canada, France, Germany, Italy, Japan, Poland, Spain, Sweden, UK, USA	France, Germany, UK
Amyloid at baseline	Not required	Not required	Required	Not required
Study Length	104 weeks treatment	80 weeks	76 weeks treatment	18 months
Disease stage(s) considered for analyses	MCI due to AD	Mild AD dementia	Mild AD dementia	Mild AD dementia
	Mild AD dementia	Moderate AD dementia

Since the study designs of EXPEDITION-1 and -2 were almost identical, data have been pooled; this is in line with prior publications. AD, Alzheimer’s disease; ADAS-Cog, Alzheimer’s disease Assessment Scale - Cognitive subscale; ADCS-ADL, Alzheimer’s Disease Cooperative Study - activities of daily living; MCI, mild cognitive impairment; NCT, National Clinical Trial.

Using data from these four data sets (GERAS, EXP1+2, EXPEDITION-3, and AMARANTH), a total of 6 cohorts, defined based on study and disease stage (MCI due to AD, mild AD dementia, moderate AD dementia) were evaluated: AMARANTH provided two cohorts (MCI due to AD and mild AD dementia); EXP1+2 provided two cohorts (mild AD dementia and moderate AD dementia); and EXPEDITION-3 and GERAS each provided one cohort (mild AD dementia). Including these six cohorts ensured multiple stages across the disease continuum (MCI due to AD through moderate AD dementia) were evaluated and data from both clinical trials and real-world studies were represented.

All studies included in these analyses were performed in accord with the ethical standards of the Committee on Human Experimentation of the institution in which the experiments were done or in accord with the Helsinki Declaration of 1975.

### Integrated Alzheimer’s Disease Rating Scale (iADRS)

The iADRS (score range, 0–144) is a linear com-bination of its two components, the ADAS-Cog_13_ (range, 0–85; higher scores indicating greater deficit of global cognition) [23] and the ADCS-iADL (instrumental items of the ADCS-ADL scale [items 6a and 7–23]; range, 0–59; lower scores indicating greater impairment) [24, 25]. Since worse outcomes are indicated by higher scores on the ADAS-Cog_13_ but lower scores on the ADCS-iADL, the ADAS-Cog_13_ score is multiplied by –1 when calculating the iADRS score; a lower iADRS score thus indicates greater impairment. A change in iADRS was considered as the change from baseline to ∼18 months (Week 76 was used for EXP1+2, Week 78 for AMARANTH, and Week 80 for EXPEDITION-3 and GERAS).

### Health outcome measures

#### Caregiver time

Caregiver time assisting study participants with basic and instrumental activities of daily living (ADLs, hours/month) was collected in all studies using the Resource Utilization in Dementia - Lite version (RUD-Lite) [26]. The RUD-Lite is designed to assess the healthcare resource utilization of patients and their caregivers and to determine the level of formal and informal care attributable to AD. Information on both the caregiver (caregiving time and work status) and participant (accommodation and healthcare resource utilization) is collected through a structured interview with the caregiver. Caregivers are asked to provide data on time spent assisting participants with basic Activities of Daily Living (bADLs), such as using the bathroom, eating, bathing, and dressing; with instrumental ADLs (iADLs) such as housework, shopping, medication use, and managing finances; and providing supervision (ensuring safety). Additional information on the calculation of caregiver time is provided in the GERAS study publication [17].

#### Costs

Cost data were collected in GERAS, using the Resource Utilization in Dementia - Lite version (RUD-Lite) [26]. Total societal cost was computed as the sum of patient direct medical costs (medication, hospitalization, emergency room visits, and outpatient visits), patient direct non-medical costs (housing, including structural adaptations to housing, community care services, consumables, and financial support received), and caregiver informal care costs (costs of caregiver time and of the caregiver missing work).

Using GERAS data, the total societal costs (euro/month) and the subsets of patient direct medical costs and patient direct non-medical costs (euro/month) were assessed. GERAS cost data were collected in 2010; to convert to 2022 costs, values were multiplied by 1.19 (inflation factor 2010 to 2022, weighted average for countries represented in GERAS) [27].

Additional information on the calculation of costs is provided in the GERAS study publication [17].

#### Caregiver burden

Caregiver burden was assessed using the Zarit Burden Interview (ZBI) (in GERAS only) and the Neuropsychiatric Inventory Caregiver Distress (NPI-D) scale (in GERAS and the EXPEDITION trials).

The ZBI [28, 29] is used to assess caregiving burden, with questions relating to burden in the relationship, emotional well-being, social and family life, finance, and loss of control over one’s life. The questionnaire is comprised of 22 items, each rated on a 5-point Likert scale, and the total score (range 0–88) is the sum of the individual item scores, with higher scores indicating greater burden.

The NPI-D [30] is a quantitative measure of the distress experienced by caregivers in relation to the individual symptom domains assessed by the NPI, a validated clinical rating instrument designed to assess patients’ psychiatric and behavioral symptoms. Twelve individual behavioral domains are examined including delusions, agitation/aggression, apathy/indifference, irritability/lability, and night-time behavioral disturbances. For the NPI-D, each domain is rated for level of caregiver distress (0–5), and the total score (range 0–60) is the sum of the individual domain scores, with higher scores indicating greater caregiver distress associated with behavioral disturbances.

#### Patient quality of life

Overall quality of life was assessed using the European Quality of Life Five Dimension (EQ-5D) and Quality of Life in Alzheimer’s Disease (QOL-AD) scale (in the EXPEDITION trials only).

The EQ-5D [31] is a non-disease specific quality of life (QoL) questionnaire widely used across disease states. It is a self-completed questionnaire in two parts, the descriptive system and the EQ visual analogue scale (EQ VAS). The descriptive system comprises five dimensions for the measurement of health-related quality of life, and each dimension is scored at 1 of 3 levels (no problems, some problems, extreme problems) for the 3L version (EQ-5D-3L) and 1 of 5 levels (no problems, slight problems, moderate problems, severe problems, and extreme problems) for the 5L version (EQ-5D-5L).

The EQ VAS records the respondent’s self-rated health on a vertical, visual analogue scale where the endpoints are labelled ‘Best imaginable health state’ and ‘Worst imaginable health state’. This information can be used as a quantitative measure of health as judged by individual respondents. In GERAS and EXPEDITION-1, -2 and -3, the caregiver version of EQ-5D-3L was employed, while in AMARANTH the EQ-5D-5L patient version was used.

The QOL-AD [32, 33] is a validated, disease-specific measure of quality of life for an AD population. The questionnaire is completed by the participant and by the caregiver (proxy); each version comprises 13 items, including questions on various QoL domains, including mood, relationships, memory, and finance. Each domain is rated on a 4-point scale, and the total score (range 13–52) is the sum of the individual domain scores, with higher scores indicating a better QoL.

For those measures described above that were captured in more than one study, the same scale version, training, and questioning format were used across studies.

### Statistical analysis

Participant and caregiver characteristics at baseline for each cohort were summarized using descriptive statistics, based on non-missing observations, and presented as mean and standard deviation (SD) for continuous variables and number of events with percentages for categorical variables.

Analysis of covariate (ANCOVA) models were used to estimate the relationship between change on the iADRS and change in the health outcome measures (change defined as change from baseline to ∼18 months). Each of the six cohorts, defined based on study and disease stage (AMARANTH - MCI due to AD and mild AD dementia cohorts; EXP1+2 - mild AD dementia and moderate AD dementia cohorts; EXPEDITION-3 - mild AD dementia cohort; and GERAS mild AD dementia cohort), were analyzed in individual models. Each model included the covariates of baseline score, country, age at baseline, caregiver relationship, and standard-of-care medication. The change from baseline regression coefficients (and 95% confidence intervals) for the iADRS derived from the models were used to compute the impact of natural disease progression and impact of disease-modifying treatment on health outcomes.

To evaluate the impact of natural disease progression on health outcomes, the 18-month change on the iADRS was approximated based on placebo data from Lilly-sponsored AD treatment trials [5, 6, 18, 34] as 7 points for MCI due to AD, 14 points for mild AD dementia, and 21 points for moderate AD dementia. Caregiver time spent and costs incurred over 18 months were calculated using the area of a triangle formula: 0.5×18×(regression coefficient from the respective ANCOVA)×(iADRS change from baseline to 18 months estimate). For health outcome scales (ZBI, NPI-D, EQ-5D, and QOL-AD), changes over 18 months were calculated by multiplying the regression coefficient by the iADRS change from baseline to 18 months estimate.

To determine the impact that a disease-modifying treatment could have on health outcome measures, we estimated the 18-month change on the iADRS with active treatment (assuming various assumptions of slowing of decline versus placebo, 20–30%), and subsequently calculated the difference between no treatment (placebo) and active treatment in effect on health outcome measure at 18 months). For illustrative purposes, we present the caregiver supervision time findings for a mild AD dementia population, in the context of “savings” (defined as the degree to which a disease-modifying treatment reduces supervision time).

To understand whether cognition and/or function (measured by the ADAS-Cog_13_ and ADCS-iADL, respectively) was the underlying explanation of any association between iADRS and health outcome changes, additional ANCOVAs were conducted. For each outcome in each cohort, a base regression model (described above) was used, and the following covariates were included and compared via the Akaike information criterion (AIC): ADCS-iADL change score, ADAS-Cog_13_ change score, both the ADCS-iADL change score and ADAS-Cog_13_ change score, and iADRS change score. A lower AIC indicated a better model fit. The significance values for each of these coefficients are also reported from the models.

All analyses were conducted at the two-sided 0.05 significance level. SAS Version 9.4 was used for all analyses.

## RESULTS

Baseline characteristics and change from baseline scores on the clinical endpoints and health outcome measures are shown by cohort in Tables 2A and B.

**Table 2A:** Characteristics of analysis populations at baseline^*^

	MCI due to AD	Mild AD dementia				Moderate AD dementia
	AMARANTH (N = 287)	EXP1+2^†^ (N = 663)	EXP-3 (N = 1,072)	AMARANTH (N = 453)	GERAS (N = 567)	EXP 1+2^†^ (N = 359)
Age (y)	72.2 (6.1)	73.3 (8.2)	73.3 (8.0)	71.0 (7.3)	77.3 (6.9)	73.7 (8.1)
Region, *n* (%)
–North America	100 (35)	298 (45)	607 (57)	142 (31)	0	151 (42)
–EU	134 (47)	180 (27)	364 (34)	248 (55)	566 (100)	95 (27)
–Other	53 (19)	185 (28)	101 (9.4)	63 (13.9)	0	113 (32)
Spouse as caregiver, *n* (%)	200 (70)	418 (63)	714 (67)	328 (72)	399 (71)	213 (59)
Medications, *n* (%)
–AChEI monotherapy	132 (46)	380 (57)	637 (59)	361 (80)	413 (73)	186 (52)
–Memantine monotherapy	0	45 (6.8)	44 (4.1)	0	40 (7.1)	23 (6.4)
–AChEI-memantine	0	162 (24)	180 (17)	2 (0.4)	26 (4.6)	124 (35)
MMSE	25.1 (2.6)	22.9 (1.9)	23.0 (2.0)	23.0 (2.2)	23.3 (1.6)	17.4 (1.1)
ADAS-Cog_13_	25.1 (7.5)	29.3 (8.6)	29.5 (8.3)	30.8 (7.5)	29.7 (7.4)	41.7 (10)
ADCS-iADL	51.4 (5.6)	46.7 (8.9)	48.9 (7.8)	47.2 (7.6)	49.5 (5.8)	39.2 (11)
iADRS	111.4 (10)	102.6 (14)	104.4 (14)	101.5 (12)	106.0 (10)	83.0 (17)

^*^Mean (standard deviation) unless otherwise stated. Tabulated findings are specific to the populations included in the current analyses and may differ from the findings published in the primary disclosures. ^†^Pooled data. AChEI, acetylcholinesterase inhibitor; AD, Alzheimer’s disease; ADAS-Cog, Alzheimer’s disease Assessment Scale –Cognitive subscale; ADCS-iADL, Alzheimer’s Disease Cooperative Study - instrumental activities of daily living; EXP, EXPEDITION; iADRS, integrated Alzheimer’s Disease Rating Scale; MCI, mild cognitive impairment; MMSE, Mini-Mental State Examination; N, total number of patients; n, number of patients.

**Table 2B:** Characteristics of analysis populations –change from baseline to 18 months for clinical endpoints and health outcome measures^*^

	MCI due to AD	Mild AD dementia				Moderate AD dementia
	AMARANTH (N = 287)	EXP1+2^*^ (N = 663)	EXP-3 (N = 1072)	AMARANTH (N = 453)	GERAS (N = 567)	EXP1+2^*^ (N = 359)
Clinical endpoints
ADAS-Cog_13_	4.3 (7.9)	6.0 (10)	6.8 (9.9)	7.3 (8.3)	5.3 (8.4)	10.2 (11)
ADCS - iADL	–3.2 (6.2)	–5.7 (9.4)	–5.9 (8.3)	–7.1 (9.6)	–3.2 (5.2)	–6.9 (9.3)
iADRS	–7.6 (12)	–11.2 (17)	–12.0 (15)	–14.1 (15)	–7.4 (10)	–15.9 (16)
Health outcome measures
Total societal cost, € /m^‡^	–	–	–	–	338 (2041)	–
Patient medical cost, € /m^‡^	–	–	–	–	–9.6 (1064)	–
Patient non-medical cost, € /m^‡^	–	–	–	–	241 (998)	–
Total caregiver time, h/m	18.9 (81.9)	27.4 (120.5)	26.4 (113.1)	18.4 (104.9)	77.5 (207.3)	41.4 (181.1)
Supervision time, h/m	10.4 (66.4)	30.5 (135.5)	24.7 (109.4)	28.0 (120.1)	63.1 (192.2)	50.4 (192.4)
ZBI	–	–	–	–	5.4 (11.7)	–
NPI-D	–	0.6 (6.5)	1.3 (6.0)	–	1.8 (7.1)	2.4 (7.7)
EQ-5D	0 (0.1)	0 (0.2)	–0.1 (0.2)	0 (0.2)	–0.1 (0.3)	–0.1 (0.3)
QOL-AD, caregiver (proxy for patient)	–	–1.7 (5.1)	–2.2 (5.1)	–	–	–2.8 (5.4)
QOL-AD, self	–	–1.1 (4.9)	–0.7 (4.5)	–	–	–0.5 (5.0)

^*^Mean (SD) unless otherwise stated. Tabulated findings are specific to the populations included in the current analyses and may differ from the findings published in the primary disclosures. ^†^Pooled data. ^‡^GERAS cost data were collected in 2010; values presented in the table have been converted to 2022 costs (multiplied by 1.19, see text for details). AD, Alzheimer’s disease; ADAS-Cog, Alzheimer’s disease Assessment Scale –Cognitive subscale; ADCS-iADL, Alzheimer’s Disease Cooperative Study –instrumental activities of daily living; EXP, EXPEDITION; EQ-5D, European Quality of Life Five Dimension; iADRS, integrated Alzheimer’s Disease Rating Scale; MCI, mild cognitive impairment; N, total number of patients; NPI-D, Neuropsychiatric Inventory Caregiver Distress; QOL-AD, Quality of Life in Alzheimer’s Disease; SD, standard deviation; ZBI, Zarit Burden Interview.

Table 3 shows regression coefficients for change from baseline for iADRS and health outcome measures by cohort. As an example, in individuals with mild dementia due to AD, a 1-point worsening on the iADRS over 18 months was associated with an increased caregiver supervision time of 1.8–2.6 hours/month and a 0.35-point worsening on the ZBI. Regression coefficients were significant (*p* < 0.05) for all populations and outcomes, with the exception of the EQ-5D in the MCI due to AD population, patient medical cost in the mild AD dementia population, and QOL-AD in moderate AD dementia population. All associations were in the expected direction of worsening (a decline of the iADRS was associated with increased costs or supervision time, for example).

**Table 3:** Change from baseline regression coefficients (95% confidence interval) for iADRS and health outcome measures by cohort

	MCI due to AD	Mild AD dementia				Moderate AD dementia
	AMARANTH (N = 287)	EXP 1+2^*^ (N = 663)	EXP-3 (N = 1072)	AMARANTH (N = 453)	GERAS (N = 567)	EXP 1+2^*^ (N = 359)
Total societal cost (€ /m)	–	–	–	–	22.1 (11.0–33.2)	–
Patient medical cost (€ /m)	–	–	–	–	0.32 (–3.6–4.2)^ns^	–
Patient non-medical cost (€ /m)	–	–	–	–	3.3 (0.16–6.53)	–
Total caregiver time (h/m)	1.8 (0.7–3.0)	1.8 (1.2–2.4)	1.9 (1.6–2.3)	0.93 (0.20–1.7)	4.3 (2.3–6.4)	2.5 (1.2–3.7)
Supervision time (h/m)	2.3 (1.3–3.2)	2.1 (1.5–2.8)	1.8 (1.4–2.3)	2.6 (1.6–3.7)	1.8 (0.025–3.6)	2.7 (1.2–4.2)
ZBI	–	–	–	–	0.35 (0.21–0.48)	–
NPI-D	–	0.12 (0.09–0.15)	0.09 (0.07–0.12)	–	0.16 (0.07–0.24)	0.12 (0.06–0.18)
EQ-5D	–0.001 (–0.002–0.001)^ns^	–0.004 (–0.005––0.003)	–0.003 (–0.004––0.003)	–0.002 (–0.004––0.001)	–0.004 (–0.006––0.001)	–0.005 (–0.006––0.003)
QOL-AD, caregiver (proxy for patient)	–	–0.11 (–0.13––0.09)	–0.09 (–0.11––0.07)	–	–	–0.09 (–0.13––0.05)
QOL-AD, self	–	–0.05 (–0.08––0.03)	–0.05 (–0.07––0.03)	–	–	0.006 (–0.03–0.05)^ns^

^*^Pooled data. Analysis of covariance models used; the coefficients are based on a 1-point decline in iADRS score. AD, Alzheimer’s disease; EQ-5D, European Quality of Life Five Dimension; EXP, EXPEDITION; iADRS, integrated Alzheimer’s Disease Rating Scale; MCI, mild cognitive impairment; N, total number of patients; NPI-D, Neuropsychiatric Inventory Caregiver Distress; ns, not significant; QOL-AD, Quality of Life in Alzheimer’s Disease; ZBI, Zarit Burden Interview (measures caregiver burden).

The degree to which the change on the iADRS with natural disease progression is associated with change on the health outcome measures is shown in Table 4; findings are based on the expected 18-month change on the iADRS (7, 14, and 21 points for MCI due to AD, mild AD dementia, and moderate AD dementia, respectively) and the coefficients shown in Table 3. As an example, in individuals with mild dementia due to AD, a 14-point decline in iADRS over 18 months is associated with a 227- to 328-hour increase in caregiver supervision time and a 4.9-point worsening on the ZBI (measuring caregiver burden) at the 18-month time point.

**Table 4:** Impact of natural disease progression (measured by change on iADRS) on health outcome measures over 18 months, using placebo data imputation

	MCI due to AD	Mild AD dementia				Moderate AD dementia
	AMARANTH (N = 287)	EXP1+2^*^ (N = 663)	EXP-3 (N = 1,072)	AMARANTH (N = 453)	GERAS (N = 567)	EXP1+2^*^ (N = 359)
Total societal cost (€)	–	–	–	–	3,314	–
Patient medical cost (€)	–	–	–	–	ns	–
Patient non-medical cost (€)	–	–	–	–	495	–
Total caregiver time (h)	113	227	239	117	542	473
Supervision time (h)	145	265	227	328	227	510
ZBI	–	–	–	–	4.9	–
NPI-D		1.68	1.26	–	2.24	2.52
EQ-5D	ns	–0.056	–0.042	–0.028	–0.056	0.105
QOL-AD, caregiver (proxy for patient)	–	–1.54	–1.26	–	–	–1.26
QOL-AD, self	–	–0.70	–0.70	–	–	ns

^*^Pooled data. For cost and time outcomes, the predicted impact was calculated using the area of a triangle formula: 0.5×18×(change from baseline regression coefficient)×(18-month change on iADRS), where change on iADRS is estimated to be 7 points for MCI, 14 points for mild AD, and 21 points for moderate AD. For scales, the predicted impact was (change from baseline regression coefficient)×(18-month change on iADRS) (see Methods for details). GERAS cost data were collected in 2010; values presented in the table have been converted to 2022 costs (multiplied by 1.19, see text for details). AD, Alzheimer’s disease; EQ-5D, European Quality of Life Five Dimension; EXP, EXPEDITION; iADRS, integrated Alzheimer’s Disease Rating Scale; MCI, mild cognitive impairment; N, total number of patients; NPI-D, Neuropsychiatric Inventory Caregiver Distress; ns, not significant; QOL-AD, Quality of Life in Alzheimer’s Disease; ZBI, Zarit Burden Interview (measures caregiver burden).

We evaluated the potential impact of a disease-modifying treatment on slowing of disease progression using caregiver time as an outcome example. Predicted savings due to disease-modifying treatment (versus no treatment) vary as a function of assumed slowing of decline in iADRS due to treatment (20–30%) and on the database evaluated and are shown in Table 5.

**Table 5:** Predicted effect of disease-modifying treatment on caregiver supervision time over 18 months for individuals with mild AD dementia

% Slowing of decline with treatment	iADRS change^*^	Caregiver supervision time^†^	Savings^‡^
20%	11.2 points	181–262	46–66 h
25%	10.5 points	170–246	57–82 h
30%	9.8 points	159–229	68–99 h

^*^Based on imputed 14-point decline in iADRS over 18 months with placebo. ^†^0.5×18×regression coefficient×iADRS change, where the regression coefficient ranged from 1.8–2.6 across all mild AD dementia cohorts (Table 3). ^‡^Calculated as caregiver supervision time estimate with natural disease progression (Table 4) minus that with disease modification (3rd column in this table). AD, Alzheimer’s disease; iADRS, integrated Alzheimer’s Disease Rating Scale.

Further modeling analyses were performed to understand which iADRS component, cognition (measured by ADAS-Cog_13_) or function (measured by ADCS-iADL), or both, was explaining the observed associations. Table 6 summarizes the results of these models and based on the AIC model fit criteria, the domain with strongest association with the outcome measure is shown. If the model including only function results in the best model fit, then function has the strongest association with the outcome of interest; where the best fitting model includes both cognition and function, then both are independently contributing to the change in outcome. The table also highlights where the iADRS scale was significant when fitted independently.

**Table 6:** Role of cognition and function in explaining any association between iADRS and health outcome changes –Findings from ANCOVA models

	MCI due to AD	Mild AD dementia				Moderate AD dementia
	AMARANTH (N = 287)	EXP 1+2^*^ (N = 663)	EXP-3 (N = 1072)	AMARANTH (N = 453)	GERAS (N = 567)	EXP 1+2^*^ (N = 359)
Total societal cost, € /m	^–^	–	–	–	FUNC^†^	–
Patient medical cost, € /m	^–^	–	–	–	ns	–
Patient non-medical cost, € /m	–	–	–	–	COG^†^	–
Total caregiver time, h/m	FUNC^†^	FUNC^†^	COG+FUNC^†^	FUNC†	FUNC^†^	COG^†^
Supervision time (h/m)	COG+FUNC^†^	COG+FUNC^†^	COG+FUNC^†^	COG†	FUNC^†^	ns^†^
ZBI	–	–	–	–	COG+FUNC^†^	–
NPI-D	–	COG+FUNC^†^	COG+FUNC^†^	–	FUNC^†^	FUNC^†^
EQ-5D	ns	COG+FUNC^†^	FUNC^†^	FUNC†	ns^†^	COG^†^
QOL-AD, caregiver (proxy for patient)	–	COG+FUNC^†^	FUNC^†^	–	–	COG+FUNC^†^
QOL-AD, self	–	FUNC^†^	COG^†^	–	–	ns

Findings from models in which ADAS-Cog_13_ (measuring cognition, COG) and ADCS-iADL (measuring function, FUNC) are fitted as explanatory variables. Significant (*p* < 0.05) drivers in the model are shown in each cell (decline in COG and/or FUNC). ^*^Pooled data. ^†^iADRS was significant when it replaced ADAS-Cog_13_ and ADCS-iADL in the model. AD, Alzheimer’s disease; EQ-5D, European Quality of Life Five Dimension; ADAS-Cog, Alzheimer’s disease Assessment Scale - Cognitive subscale; ADCS-iADL, Alzheimer’s Disease Cooperative Study - instrumental activities of daily living; ANCOVA, Analysis of covariate; COG, cognition; EXP, EXPEDITION; FUNC, function; iADRS, integrated Alzheimer’s Disease Rating Scale; MCI, mild cognitive impairment; N, total number of patients; NPI-D, Neuropsychiatric Inventory Caregiver Distress; ns, not significant; QOL-AD, Quality of Life in Alzheimer’s Disease; ZBI, Zarit Burden Interview (measures caregiver burden).

When both cognition and function were fitted in the same model, the one which showed the greatest explanatory power was dependent upon the study, disease stage, and outcome. As an example, for total caregiver time, functional decline was the driver of change in this outcome in the MCI due to AD and, in most cases, the mild AD dementia populations, while cognitive decline was the driver in the moderate AD dementia population for this variable. For supervision time, results were more variable, with either decline in cognition alone, function alone or cognition *and* function driving the findings in mild AD dementia populations, cognition and function driving the findings in MCI due to AD, and neither cognitive nor functional decline driving the findings in the moderate AD dementia population. For all outcomes, when the two individual variables were replaced with the iADRS, associations with the outcome were significant in all cases.

## DISCUSSION

This retrospective analysis explored the relationship between change on the iADRS and changes in health outcome measures across the AD continuum (MCI due to AD to moderate AD dementia), using data from five studies.

Across disease stages and studies, a change (decline) on the iADRS over 18 months was statistically significantly associated with changes on health outcome measures in most cases. Findings were generally consistent across the studies, although in the case of GERAS (the only observational study) total caregiver time was somewhat higher than for the clinical trials and there was generally more uncertainty (wider confidence intervals) around the regression point estimates. Whether this reflects a more heterogeneous population in GERAS and/or a different enrollment strategy for the observational study needs further clarification.

The public health impact of AD is well recognized, with the long duration of the illness translating to an extended period in a state of disability and dependence [35]. Individuals with AD are often cared for in their homes by family or friends, and the cost of AD and related dementias translates to more than 11 million Americans providing more than 15 billion hours of unpaid care per year. Moreover, the demands placed on the caregiver result in a greater risk for anxiety, depression, and poorer quality of life than caregivers of people with other conditions. In an analysis of baseline data from the GERAS study, a greater AD severity was associated with both a greater caregiver burden (subjective measure) and overall caregiver time; caregiver burden and supervision time were greater with worse patient functioning (on instrumental activities of daily living) [14]. In a further analysis of the GERAS data, both cognitive and functional decline were associated with increases in costs and caregiver burden in patients with mild AD dementia [36].

Establishing a relationship between the outcome measure(s) used in clinical trials (e.g., iADRS) and important patient/caregiver variables that are not otherwise measured (e.g., caregiver burden/time) could permit the utilization of the former as a ‘surrogate marker’ for the latter. Assuming such a relationship exists, one may be able to take this a step further and predict that an effect of a disease-modifying treatment (DMT) on disease progression (as measured by change in iADRS score) could translate to an effect on patient/caregiver variables.

Caregiver time has been shown to be associated with the performance on the ADAS-Cog, a direct clinical trial outcome [37]. Our findings demonstrate a significant association between disease progression as measured by the iADRS and caregiver outcomes across the disease continuum. Based on the findings presented, it can be predicted that for an individual with MCI due to AD, an expected decline of 7 points on the iADRS over 18 months will result in 113 hours increase in caregiver time over the same period. For individuals who have progressed to mild AD dementia, an expected decline of 14 points on the iADRS over a further 18 months will result in 117–542 (depending upon study cohort) hours of caregiver time). In the moderate dementia stage of AD, caregiver time spent will be approximately 473 hours over 18 months (based on a 21-point decline on the iADRS). A DMT that slows disease progression could result in savings in caregiver time. In the example provided in Table 5, depending on the degree to which a DMT slows disease progression, its use could result in a savings of 46–99 hours of caregiver time over 18 months. Since a DMT slows disease progression and changes the slope of the disease trajectory, one can expect that follow up after 18 months would result in an even greater savings in caregiver time. These numbers pertain to only *one* affected individual. Given that dementia due to AD currently affects more than 6 million individuals in the US [35], one can appreciate the significant impact to society. Using the data presented here, over 18 months, a 14-point change on the iADRS translates to ∼700 million to 3 billion hours of caregiver time; this is a conservative estimate based on the assumption of the 6 million affected individuals having only mild AD dementia. We can take this a step further and estimate the savings in caregiver time with treatment using a DMT; using the most conservative assumption of 20% slowing of disease, a DMT would result in a 270 to 396 million hours savings in caregiver time over 18 months.

Differences in findings across the six cohorts in this study could be attributed to differences between disease severities or other differences between the trials. The EXP1+2 data provide the opportunity to compare findings across different stages of the disease *within* a trial. The regression coefficients for the mild and moderate AD dementia cohorts of EXP1+2 were similar (suggesting that the a 1-point change on the iADRS will translate to the same increase in caregiver time in these populations). However, the rate of disease progression is greater in the moderate AD dementia population, such that, as the disease becomes more advanced, caregiver time will be greater. This finding is in agreement with previous published findings discussed above [14]. This methodology could be similarly applied to other outcomes to understand the implication of treatment on health outcomes, via its effect of change on iADRS.

Caregiver burden was measured using the ZBI (GERAS only) and NPI-D (EXPEDITION trials only); burden generally increased with disease progression. As for caregiver time, within trial comparison of caregiver burden across disease severities was feasible using the EXP1+2 database; the higher NPI-D regression coefficient for the moderate AD dementia cohort versus the mild AD dementia cohort, such that a 1-point change on the iADRS in mild AD dementia translates to less additional caregiver burden compared with a 1-point change in moderate AD dementia.

A decline on the iADRS was also associated with worsening in measures of participant quality of life. While EQ-5D scores did not show meaningful change with change on the iADRS in the MCI due to AD population, it should be noted that the EQ-5D is a generic HR-QoL tool that was not designed specifically for patients with dementia. The EQ-5D is predominantly focused on the assessment of functional and emotional impairments, and the absence of a specific cognitive domain may explain why the EQ-5D detects less marked differences between mild and moderate cognitive impairments. In the case of the QOL-AD, the change from baseline in the caregiver-rated score was higher than for the participant-rated score. As a result, a 1-point change on the iADRS was associated with a greater decline on the QOL-AD when rated by the caregiver versus participant. This discrepancy in scores increased with increased disease severity. These findings likely reflect a caregiver’s greater awareness of the impact of changes in a patient’s cognitive and functional abilities on the patient’s QoL, with caregiver and patients scores diverging with increasing disease severity [38]. More research is needed to fully understand what drives patient QoL and how to best measure it in patients with AD.

GERAS data pertaining to costs of AD are also presented. Based on these findings, it can be predicted that for an individual with mild AD dementia, an expected decline of 14 points on the iADRS over 18 months will result in a societal cost of approximately € 3314. Again, this pertains to only one individual. Using the same assumption as for our caregiver time example above, with a 14-point decline on the iADRS over 18 months, this cost of € 3314 (∼US$3770) translates to US$22.6 billion in US societal cost.

A key attribute of the iADRS is that it measures both cognition and function. As a composite, its effect size is a function of the correlation between the components and the ratio of effect sizes of the components [3]. The components of the iADRS (ADAS-Cog_13_ and ADCS-iADL) are modestly correlated, and the effect size of the composite typically falls in between the effect sizes of its components, providing more opportunity to detect changes than the individual components alone. Support for the ability of this scale to reliably capture change across the core domains of AD is perhaps exemplified in the findings from the two identically-designed solanezumab treatment trials EXPEDITION-1 and -2 [18]. In EXPEDITION-1, the treatment effect was significant for the cognitive outcome but not the functional outcome, while in EXPEDITION-2 the functional but not cognitive outcome was significant; change on the iADRS was significant in both studies [2].

In the current study, analyses were performed to determine whether cognition and/or function was driving the association between change in iADRS and individual health outcome measures. Based on the findings in Table 6, it is evident that the driver was dependent upon the outcome. Importantly, whether cognition or function or both were the driver, the associations between change on the iADRS and change on the outcome were significant in all cases, across MCI due to AD, mild AD dementia and moderate AD dementia. That is, a change on the iADRS was associated with a change in health outcome measures, whether driven by cognition only, function only, or both cognition and function. This finding provides further support for the more comprehensive performance that a summative composite (here, iADRS) delivers versus its components [3].

There are limitations to this analysis. MCI due to AD and moderate AD dementia findings are presented for only AMARANTH and EXP1+2, respectively, while some outcome measures were included in only one study (e.g., cost findings and ZBI in GERAS only); in these cases, findings should be interpreted more cautiously. Amyloid pathology was not confirmed in all cohorts and disease course differs between those with/without amyloid. This analysis was retrospective; a prospectively designed study to specifically address clinical meaningfulness of iADRS would permit consistency in the use of health outcome measures across severity levels.

In conclusion, across the MCI due to AD, mild AD dementia and moderate AD dementia populations, a decline on the iADRS was associated with a meaningful increase in caregiver burden (time, ZBI). A decline on the iADRS was also associated with worsening in measures of participant quality of life and caregiver distress, but the associations were more modest. A change in iADRS score was predictive of outcomes that were driven by cognitive or functional decline or both.

## References

[1] Wessels AM , Andersen S , Dowsett S , Siemers E (2018) The integrated Alzheimer’s Disease Rating Scale (iADRS) findings from the EXPEDITION3 trial. J Prev Alzheimers Dis 5, 134–136.2961670610.14283/jpad.2018.10

[2] Wessels A , Siemers E , Yu P , Andersen S , Holdridge K , Sims J , Sundell K , Stern Y , Rentz D , Dubois B (2015) A combined measure of cognition and function for clinical trials: The integrated Alzheimer’s disease rating scale (iADRS). J Prev Alzheimers Dis 2, 227–241. 27019841 10.14283/jpad.2015.82 PMC4806404

[3] Liu-Seifert H , Andersen S , Case M , Sparks J , Holdridge KC , Wessels AM , Hendrix S , Aisen P , Siemers E (2017) Statistical properties of continuous composite scales and implications for drug development. J Biopharm Stat 27, 1104–1114. 28402165 10.1080/10543406.2017.1315819

[4] Mintun MA , Lo AC , Duggan Evans C , Wessels AM , Ardayfio PA , Andersen SW , Shcherbinin S , Sparks J , Sims JR , Brys M (2021) Donanemab in early Alzheimer’s disease. N Engl J Med 384, 1691–1704. 33720637 10.1056/NEJMoa2100708

[5] Wessels AM , Tariot PN , Zimmer JA , Selzler KJ , Bragg SM , Andersen SW , Landry J , Krull JH , Downing AM , Willis BA (2020) Efficacy and safety of lanabecestat for treatment of early and mild Alzheimer disease: The AMARANTH and DAYBREAK-ALZ randomized clinical trials. JAMA Neurol 77, 199–209. 31764959 10.1001/jamaneurol.2019.3988 PMC6902191

[6] Honig LS , Vellas B , Woodward M , Boada M , Bullock R , Borrie M , Hager K , Andreasen N , Scarpini E , Liu-Seifert H (2018) Trial of solanezumab for mild dementia due to Alzheimer’s disease. N Engl J Med 378, 321–330. 29365294 10.1056/NEJMoa1705971

[7] Biogen Update on the Phase 4 ENVISION Confirmatory Study of ADUHELM®2022. https://investors.biogen.com/news-releases/news-release-details/update-phase-4-envision-confirmatory-study-aduhelmr, Accessed February 10, 2022.

[8] ClinicalTrials.gov. Simufilam 100mgfor Mild-to-Moderate Alzheimer’s Disease (RETHINK-ALZ). https://www.clinicaltrials.gov/ct2/show/NCT04994483?term=iADRS&draw=2&rank=13, Accessed February 10, 2022.

[9] ClinicalTrials.gov. Simufilam 50 mg or 100 mg for Mild-to-Moderate Alzheimer’s Disease (REFOCUS-ALZ). https://www.clinicaltrials.gov/ct2/show/NCT05026177?term=iADRS&draw=3&rank=12, Accessed February 10, 2022.

[10] ClinicalTrials.gov. ADvance II Study: DBS-f in Patients With Mild Alzheimer’s Disease. https://www.clinicaltrials.gov/ct2/show/NCT03622905?term=iADRS&draw=2&rank=2, Accessed February 10, 2022.

[11] ClinicalTrials.gov. A Study of JNJ-63733657 in Participants With Early Alzheimer’s Disease (Autonomy). https://www.clinicaltrials.gov/ct2/show/NCT04619420?term=iADRS&draw=2&rank=1, Accessed February 10, 2022.

[12] Center for Disease Control and Prevention. Caregiving for a Person with Alzheimer’s Disease or a Related Dementia 2019. https://www.cdc.gov/aging/caregiving/alzheimer.htm, Accessed February 10, 2022.

[13] Deb A , Thornton JD , Sambamoorthi U , Innes K (2017) Direct and indirect cost of managing Alzheimer’s disease and related dementias in the United States. Expert Rev Pharmacoecon Outcomes Res 17, 189–202. 28351177 10.1080/14737167.2017.1313118 PMC5494694

[14] Haro J , Kahle-Wrobleski K , Bruno G , Belger M , Dell’Agnello G , Dodel R , Jones R , Reed C , Vellas B , Wimo A (2014) Analysis of burden in caregivers of people with Alzheimer’s disease using self-report and supervision hours. J Nutr Health Aging 18, 677–684.2522610610.1007/s12603-014-0500-x

[15] Tochel C , Smith M , Baldwin H , Gustavsson A , Ly A , Bexelius C , Nelson M , Bintener C , Fantoni E , Garre-Olmo J (2019) What outcomes are important to patients with mild cognitive impairment or Alzheimer’s disease, their caregivers, and health-care professionals? A systematic review. Alzheimers Dement (Amst) 11, 231–247. 30906845 10.1016/j.dadm.2018.12.003 PMC6411507

[16] Siemers ER , Sundell KL , Carlson C , Case M , Sethuraman G , Liu-Seifert H , Dowsett SA , Pontecorvo MJ , Dean RA , Demattos R (2016) Phase 3 solanezumab trials: Secondary outcomes in mild Alzheimer’s disease patients. Alzheimers Dement 12, 110–120. 26238576 10.1016/j.jalz.2015.06.1893

[17] Wimo A , Reed CC , Dodel R , Belger M , Jones RW , Happich M , Argimon JM , Bruno G , Novick D , Vellas B (2013) The GERAS study: A prospective observational study of costs and resource use in community dwellers with Alzheimer’s disease in three European countries–study design and baseline findings. J Alzheimers Dis 36, 385–399. 23629588 10.3233/JAD-122392

[18] Doody RS , Thomas RG , Farlow M , Iwatsubo T , Vellas B , Joffe S , Kieburtz K , Raman R , Sun X , Aisen PS (2014) Phase 3 trials of solanezumab for mild-to-moderate Alzheimer’s disease. N Engl J Med 370, 311–321. 24450890 10.1056/NEJMoa1312889

[19] ClinicalTrials.gov. Effect of LY2062430 on the Progression of Alzheimer’s Disease (EXPEDITION). https://clinicaltrials.gov/ct2/show/NCT00905372, Accessed February 10, 2022.

[20] ClinicalTrials.gov. Effect of LY2062430 on the Progression of Alzheimer’s Disease (EXPEDITION2). https://clinicaltrials.gov/ct2/show/NCT00904683, Accessed Feb-ruary 10, 2022.

[21] ClinicalTrials.gov. An Efficacy and Safety Study of Lanabecestat (LY3314814) in Early Alzheimer’s Disease (AMARANTH). https://clinicaltrials.gov/ct2/show/record/NCT02245737, Accessed February 10, 2022.

[22] ClinicalTrials.gov. Progress of Mild Alzheimer’s Disease in Participants on Solanezumab Versus Placebo (EXPEDITION 3). https://www.clinicaltrials.gov/ct2/show/study/NCT01900665, Accessed February 10, 2022.

[23] Mohs RC , Knopman D , Petersen RC , Ferris SH , Ernesto C , Grundman M , Sano M , Bieliauskas L , Geldmacher D , Clark C (1997) Development of cognitive instruments for use in clinical trials of antidementia drugs: Additions to the Alzheimer’s Disease Assessment Scale that broaden its scope. Alzheimer Dis Assoc Disord 11, S13–S21. 9236948

[24] Galasko D , Bennett D , Sano M , Ernesto C , Thomas R , Grundman M , Ferris S (1997) An inventory to assess activities of daily living for clinical trials in Alzheimer’s disease. Alzheimer Dis Assoc Disord 11(Suppl 2), S33–S39. 9236950

[25] Galasko D , Kershaw PR , Schneider L , Zhu Y , Tariot PN (2004) Galantamine maintains ability to perform activities of daily living in patients with Alzheimer’s disease. J Am Geriatr Soc 52, 1070–1076. 15209643 10.1111/j.1532-5415.2004.52303.x

[26] Wimo A WB (2003) Resource Utilization in Dementia: RUD Lite. Brain Aging 3, 48–59.

[27] Inflation Tool calculator. https://www.inflationtool.com/, Accessed February 10, 2022.

[28] Bachner Y , O’Rourke N (2007) Reliability generalization of responses by care providers to the Zarit Burden Interview. Aging Mental Health 11, 678–685. 18074255 10.1080/13607860701529965

[29] Zarit S , Zarit J (1987) *Instructions for the burden interview*. Pennsylvania State University, University Park.

[30] Kaufer DI , Cummings JL , Christine D , Bray T , Castellon S , Masterman D , MacMillan A , Ketchel P , DeKosky ST (1998) Assessing the impact of neuropsychiatric symptoms in Alzheimer’s disease: The Neuropsychiatric Inventory Caregiver Distress Scale. J Am Geriatr Soc 46, 210–215. 9475452 10.1111/j.1532-5415.1998.tb02542.x

[31] EQ-5D instrument, https://euroqol.org/eq-5d-instruments/, Accessed October 2021.

[32] Logsdon RG , Gibbons LE , McCurry SM , Teri L (2002) Assessing quality of life in older adults with cognitive impairment. Psychosomatic Med 64, 510–519. 10.1097/00006842-200205000-00016 12021425

[33] Thorgrimsen L , Selwood A , Spector A , Royan L , de Madariaga Lopez M , Woods R , Orrell M (2003) Whose quality of life is it anyway? The validity and reliability of the Quality of Life-Alzheimer’s Disease (QoL-AD) scale. Alzheimer Dis Assoc Disord 17, 201–208. 14657783 10.1097/00002093-200310000-00002

[34] Doody RS , Raman R , Farlow M , Iwatsubo T , Vellas B , Joffe S , Kieburtz K , He F , Sun X , Thomas RG (2013) A phase 3 trial of semagacestat for treatment of Alzheimer’s disease. N Engl J Med 369, 341–350. 23883379 10.1056/NEJMoa1210951

[35] Alzheimer’s Association. 2021 Alzheimer’s Disease Fact and Figures. https://www.alz.org/media/Documents/alzheimers-facts-and-figures.pdf, Accessed February 11, 2022.

[36] Jones RW , Lebrec J , Kahle-Wrobleski K , Dell’Agnello G , Bruno G , Vellas B , Argimon JM , Dodel R , Haro JM , Wimo A (2017) Disease progression in mild dementia due to Alzheimer disease in an 18-month observational study (GERAS): The impact on costs and caregiver outcomes. Dement Geriatr Cogn Disord Extra 7, 87–100. 10.1159/000461577 PMC5465649 28611822

[37] Clipp EC , Moore MJ (1995) Caregiver time use: An outcome measure in clinical trial research on Alzheimer’s disease. Clin Pharmacol Ther 58, 228–236. 7648773 10.1016/0009-9236(95)90201-5

[38] Landeiro F , Mughal S , Walsh K , Nye E , Morton J , Williams H , Ghinai I , Castro Y , Leal J , Roberts N (2020) Health-related quality of life in people with predementia Alzheimer’s disease, mild cognitive impairment or dementia measured with preference-based instruments: A systematic literature review. Alzheimers Res Ther 12, 154. 33208190 10.1186/s13195-020-00723-1 PMC7677851

